# Elucidating the Performance Limitations of Lithium-ion Batteries due to Species and Charge Transport through Five Characteristic Parameters

**DOI:** 10.1038/srep32639

**Published:** 2016-09-07

**Authors:** Fangming Jiang, Peng Peng

**Affiliations:** 1 Laboratory of Advanced Energy Systems, Guangdong Key Laboratory of New and Renewable Energy Research and Development, CAS Key Laboratory of Renewable Energy, Guangzhou Institute of Energy Conversion, Chinese Academy of Sciences (CAS), Guangzhou 510640, China

## Abstract

Underutilization due to performance limitations imposed by species and charge transports is one of the key issues that persist with various lithium-ion batteries. To elucidate the relevant mechanisms, two groups of characteristic parameters were proposed. The first group contains three characteristic time parameters, namely: (1) *t*_*e*_, which characterizes the Li-ion transport rate in the electrolyte phase, (2) *t*_*s*_, characterizing the lithium diffusion rate in the solid active materials, and (3) *t*_*c*_, describing the local Li-ion depletion rate in electrolyte phase at the electrolyte/electrode interface due to electrochemical reactions. The second group contains two electric resistance parameters: *R*_e_ and *R*_s_, which represent respectively, the equivalent ionic transport resistance and the effective electronic transport resistance in the electrode. Electrochemical modeling and simulations to the discharge process of LiCoO_2_ cells reveal that: (1) if *t*_*e*_, *t*_*s*_ and *t*_*c*_ are on the same order of magnitude, the species transports may not cause any performance limitations to the battery; (2) the underlying mechanisms of performance limitations due to thick electrode, high-rate operation, and large-sized active material particles as well as effects of charge transports are revealed. The findings may be used as quantitative guidelines in the development and design of more advanced Li-ion batteries.

Lithium-ion batteries have been widely applied in various portable consumer electronics. Compared with other batteries, lithium-ion batteries perform better in terms of energy-to-weight ratio, exhibit almost zero memory effect, and experience low self-discharge when in idle state^1^,^2^,^3^,^4^. Nowadays, due to its very promising perspective for uses in electric vehicles (EV), smart grids and communication base stations, lithium-ion battery possibly grows into the dominant green energy storage or supply equipment of this century.

Main factors that retard the growth of lithium-ion battery include underutilization, stress-induced material damage, capacity fade, and possible occurrence of thermal runaway^5^. Researchers have poured considerable endeavors to commercialize different types and/or chemistries of lithium-ion batteries. Selecting a lithium-ion battery for a certain application depends mainly on the chemistry of cathode and other physical factors involved in the fabrication of cells, e.g. density of the material, composition and solid particle size in electrodes, and the cell geometry. Various chemistries have been considered for the fabrication of cathode materials for lithium-ion batteries^6^,^7^,^8^,^9^,^10^,^11^. Several principal cathode materials are lithium cobalt oxide (LiCoO_2_), lithium manganese oxide (LiMn_2_O_4_) and lithium iron phosphate (LiFePO_4_). The Performance of the battery is significantly affected by the cell geometry, the cathode material and the preparation or fabrication method of cathode^12^. Improving the design of batteries to realize maximum energy and power performance requires a thorough understanding of how physical properties of electrode materials such as species diffusivity and electric conductivity, operational parameters like charge/discharge rate, and cell structural parameters like electrode thickness and particle size of solid active materials influence the cell performance.

Extensive research has been conducted. Effects of electrode thickness^13^,^14^,^15^,^16^, particle size^17^,^18^,^19^,^20^,^21^ and discharge rate^22^,^23^,^24^,^25^,^26^ on battery performance were quantified experimentally. Nagarajan *et al*.^27^ established a mathematical model for the study of particle size distribution (PSD) effects on discharge behaviors of intercalation electrode systems. Results showed that the electrode utilization could be increased when using a binary mixture of differing particle sizes. Garcia *et al*.^28^ developed a theoretical framework describing the kinetics of multiple charged species. The model results suggested controlling the transport paths to the back of the positive porous electrode, maximizing the surface area for intercalating lithium ions, and carefully controlling the spatial distribution and particle size of active materials could improve the battery performance. Golmon *et al*.^29^ found from numerical simulations that electrodes with functionally graded porosity and particle size distribution could enhance the usable energy capacity of the battery. A detailed electrochemistry model coupled with an optimization algorithm was developed to examine^30^,^31^,^32^,^33^,^34^ the effects of cycling rate and cathode solid particle size, species diffusivity, and electronic conductivity on the specific energy and specific power of battery. It was found from simulation results that the available energy decreased with a faster cycling rate, larger active material particle size, and lower species diffusivity. Doyle *et al*.^35^ found from experiments and simulations that minor solid-phase diffusion limitation effects existed in the carbon electrode and solution-phase diffusion limitations might become notably significant for a cell with an increased electrode thickness or a decreased initial salt concentration (decreased down to 1 mol•L^−1^). A later work by Doyle *et al*.^36^ derived analytical solutions, which described three performance-limiting phenomena due respectively to solution-phase diffusion, solid-phase diffusion, and charge transport. Arora *et al*.^37^ concluded from experiments and simulations that the solution-phase diffusion limitations were the major limiting factor for Li-ion polymer cells during high-rate discharges. Smith *et al*.^38^ found from numerical simulations that end of high-rate discharges might be caused by near-depletion of lithium species at negative electrode solid active material surfaces, full saturation of lithium at positive electrode solid active material surfaces, or local Li-ion depletion in the electrolyte. Hasan *et al*.^39^ investigated the cell performance and the mechanisms limiting cell performance during fast-charge operations at moderate and extreme operation temperatures by an electrochemical-thermal coupling model. It was found that appropriate electrode design, such as a reduced electrode thickness and an increased porosity in the electrode, resulted in improved rapid-charge performance. Ogihara *et al*.^40^ systematically investigated the dependence of the electrochemical behavior of intercalated LiNiO_2_-based electrode on its thickness. They found that the ion-conduction pathway in the porous electrodes strongly affected the battery power capability; thick electrodes with high-loading active materials were found to have high energy density. Zhao *et al*.^41^ investigated the impacts of electrode thickness on the electrochemical and thermal properties of lithium-ion battery cells based on physical experiments and a combination model consisting of a one-dimensional (1D) electrochemical model and a three-dimensional (3D) thermal model. They found a battery of thicker electrodes had relatively higher internal resistance, which could result in a lower power output and an earlier stop of discharge (particularly high rate discharge) due to the longer diffusion distance and more serious concentration polarization.

A better understanding of the cell design and operational parameters on its overall performance can provide guidelines and benchmarks for tailoring battery design and constituting optimized management strategy for various application circumstances. Though a great amount of work has been done, there is still a lack of general and systematical analysis on the mechanisms of transport-related performance limitations of Li-ion batteries during charge/discharge operations. This work aims to elucidate the mechanisms of battery performance being limited by species and charge transport through a general characteristic analysis method. Five parameters are proposed to characterize the involved species and charge transport during battery charge/discharge processes, and how these parameters affect the battery performance is analyzed relying on a numerical model of Li-ion battery charge/discharge processes. Particularly, the numerical study takes the discharge process of a graphite/LiPF_6_/LiCoO_2_ battery to illustrate the characteristic analysis method; cases with different cell designs and/or operational parameters including electrode thickness, discharge rate, active material particle size, and material electric conductive property are calculated.

## Characterizing Charge and Species Transport

Charge and species transport occur inside Li-ion batteries during discharge/charge processes. Charge transport includes ionic charge transport in the electrolyte and electronic charge transport in the solid phase; species transport includes Li-ion transport in the electrolyte and lithium transport in electrode active materials. These transport processes basically determine the performance of a Li-ion battery. For example, local Li-ion concentration in the electrolyte may be exhausted if the local consumption rate of Li-ions is far more than the transport rate of Li-ions in the electrolyte. And this phenomenon will lead to deteriorated performance (e.g. reduced capacity or capacity loss) of battery/cells. We characterize the species and charge transport in Li-ion battery charge/discharge processes to facilitate discussion about the mechanisms that limit the performance of Li-ion batteries.

### Species transport in the electrolyte and solid phase

During Li-ion battery discharge/charge processes, the electrochemical reaction, which consumes or generates lithium atoms or ions (Li^+^), takes place at the interface of solid active materials and electrolyte; the lithium ions are being transported in the electrolyte by molecular diffusion and electric migration; the lithium atoms are diffusing into the solid active materials or out from the active material interior to the interface of solid and electrolyte phase. The governing equation for Li^+^ species transport in the electrolyte phase is formulated as^42^:


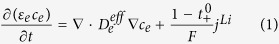


where 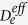 denotes the effective diffusion coefficient of Li^+^ in electrolyte phase, *c*_*e*_ is the lithium concentration in electrolyte, *ε*_*e*_ is the volume fraction of electrolyte, *t* is the time, 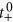 is the transference number of Li^+^ dissolved in the electrolyte, *F* is the Faraday’s constant, and *j*^*Li*^ is the transfer current density. By assuming the solid active material to be some spherical particles, the equation governing lithium species transport in solid phase can be formulated as^42^:


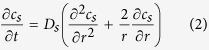


where *D*_*s*_ denotes the diffusion coefficient of lithium in the solid active material, *c*_*s*_ is the lithium concentration in solid phase, and *r* is the spherical coordinate.

The transfer current density *j*^Li^, which quantifies the electrochemical (EC) reaction rate, is normally calculated by the Butler-Volmer equation^42^:


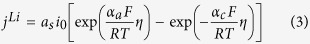


where *a*_*s*_ denotes the specific surface area, *i*_*0*_ is the exchange current density, *α*_*a*_ is the anodic transfer coefficient, *α*_*c*_ is the cathodic transfer coefficient, *R* is the universal gas constant, *T* is the temperature, and *η* is the surface overpotential.

We define three parameters to characterize the species transport processes in Li-ion batteries, i.e. *t*_*e*_, *t*_*s*_, and *t*_*c*_.


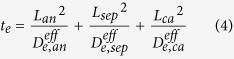



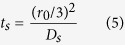



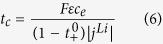


where, *L*_*an*_, *L*_*ca*_, and *L*_*sep*_ denote the thickness of anode electrode, cathode electrode and separator, respectively; *r*_*0*_ represents the radius of the spherical active material particles. In Eq. (5), the factor 1/3 is the shape factor accounting for the diffusion transport in spherical objects^43^. All the three parameters have the unit of time. Physically, *t*_*e*_ s*t*ands for a characteristic time describing the Li-ion transport rate in the electrolyte; *t*_*s*_ can be looked as a characteristic time characterizing the lithium diffusion process in the solid active materials; *t*_*c*_ is a characteristic time relating with the local Li-ion depletion rate in electrolyte at the electrolyte/electrode interface due to the EC reaction. Because of the presence of the transference coefficient (i.e. 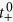) in Eq. (6), the effect of electric migration on Li-ion transport has been incorporated. We presume that there exists a relationship in-between these three parameters that can lead to the battery being of the best charge/discharge performance, without any performance-limitations due to species transport. Generally speaking, as long as the three characteristic time parameters are on the same order of magnitude, the battery should not get into any performance-limitations caused by the species transport processes.

### Charge transport in the electrolyte and solid phase

The charge transport equation for lithium-ion charge in the electrolyte phase can be expressed as^42^:


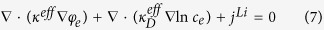


where *κ*^*eff*^ denotes the effective ionic conductivity in electrolyte, and *φ*_*e*_ is the electric potential in electrolyte. By Taylor expansion with the second and higher order terms omitted with respect to the second term on the left-hand side of Eq. (7), it yields:


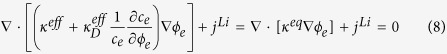


The diffusional conductivity 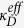
is dependent on the ionic conductivity *κ*^*eff*^, and can be calculated by virtue of concentrated solution theory^42^, as


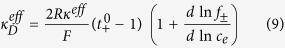


where *f*_±_ is the mean molar activity coefficient of the electrolyte.

Note that we have defined a parameter *κ*^*eq*^ in Eq. (8), which means the overall equivalent ionic conductivity in the electrolyte.

The electronic charge transport in solid phase is governed by the following conservation equation^42^:


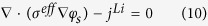


where *σ*^*eff*^ denotes the effective electronic conductivity in solid phase, and *φ*_*s*_ is the electric potential in solid phase.

We define two parameters, namely, the equivalent ionic transport resistance in the electrolyte (i.e. *R*e) and the effective electronic transport resistance in solid phase (i.e. *R*_s_), to characterize the charge transport processes in electrodes.


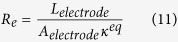



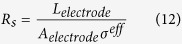


where, *L*_*electrode*_ (m) and *A*_*electrode*_ (m^2^) represent the thickness and side surface area of the electrode, respectively. Note that *L*_*electrode*_ may take different values for anode and cathode electrode. Physically, the charge transport will always seek transport paths of the least transport resistance, which may affect the electric potential (i.e. *φ*_s_ and *φ*_e_) distributions and EC reaction distribution in the corresponding electrode.

### Numerical modeling

Numerical modeling is widely used and plays an important role in the research and development of various electrochemical cells^42^,^44^,^45^,^46^. In the present work, we choose the lithium cobalt oxide battery as a template Li-ion battery and establish a three-dimensional numerical model, which describes the electrochemical-physical processes in lithium cobalt oxide batteries during charge and discharge operations. For a lithium-ion battery of different chemistry, the involved multi-physical transport basically follows the same principles of conservation^47^. Specially, we simulate discharge processes of lithium cobalt oxide batteries to study the performance limitations related to charge and species transport. We assume the battery is thermally well-managed and the whole battery remains at an optimum constant temperature (i.e. 25 °C considered in the present work) throughout the discharge process.

### Physical and mathematical model

**Figure 1:**
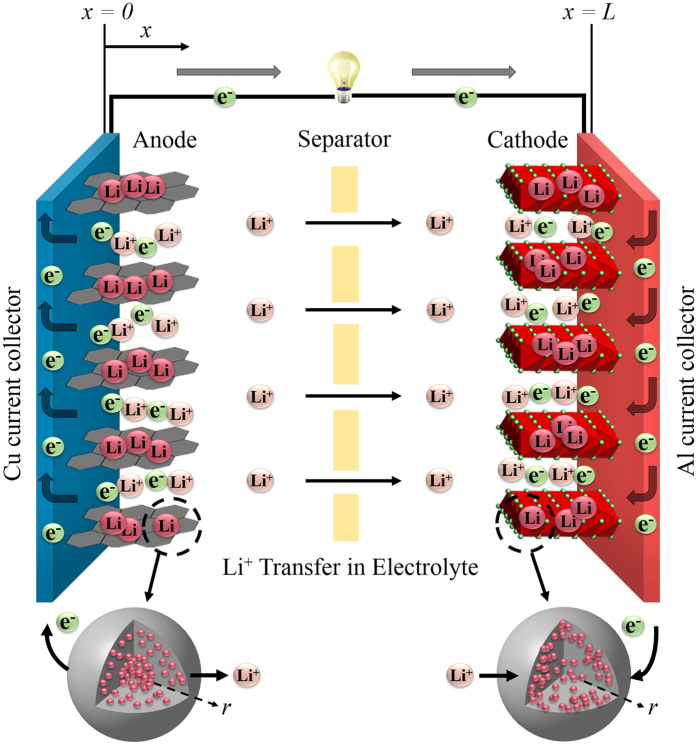
Schematic of the involved species/charge transports and electrochemical reactions in lithium-ion cells during discharge. The electrode through-plane direction is indicated by the *x* coordinate.

Figure 1 schematically shows the geometry and the involved species and charge transport during discharge of a graphite/LiPF_6_/LiCoO_2_ battery considered in the present work. The cell consists of a copper current collector (AC), a negative electrode, a separator, a positive electrode, and an aluminum current collector (CC). Both the electrodes and the separator are porous in nature. The electrode is composed of an active material, a polymer binder, some conductive filler, and the electrolyte. The active materials of anode and cathode are graphite mesocarbon microbeads and Li_y_CoO_2_, respectively. The electrolyte is lithium hexafluorophosphate (LiPF_6_) in a mixture of propylene carbonate, ethylene carbonate, and dimethyl carbonate. The porous separator is composed of the liquid electrolyte and a polymer matrix. Illustrated also in Fig. 1 is the assumed lithium diffusion model in solid particles of anode and cathode active materials when discharging the battery.

During charge/discharge processes, the EC reactions occurring in electrodes can be expressed as follows.

In the anode composite electrode,


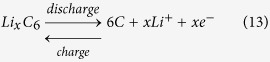


In the cathode composite electrode,


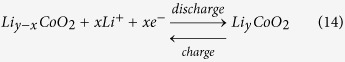


The electrochemical model for graphite/LiPF_6_/LiCoO_2_ battery developed in this work is based on porous electrode and concentrated solution theories^48^,^49^. Major assumptions include^42^ (1) Electrode active materials are assumed to be spherical particles of uniform size; (2) Side reactions are ignored and no gas phase is present during charge/discharge operations; (3) The electrolyte is a concentrated binary solution, which means that it is dissociated into a cation and an anion; (4) The transport of lithium species relies on diffusion and migration in the electrolyte solution while only on diffusion in the solid active materials; (5) EC reactions of lithium insertion and extraction processes follow the Butler-Volmer equation, i.e. Eq. (3), which describes a large class of electrode reactions; (6) Interfacial electrical and chemical equilibrium exist at the electrode/electrolyte interface; (7) The effective transport coefficients are calculated in terms of the Bruggeman theorem^50^, namely, *π*^*eff*^ = *πε*^1.5^, where *π* denote the intrinsic transport coefficient of a material and *ε* the component volume fraction of this material in the composite electrodes/separator.

In numerical modeling, we treat the battery as a single-domain of multiple sub-regions associated with different sets of physical-chemical properties. This circumvents difficulties about matching boundary conditions at the interior interfaces of the battery. The model equations together with boundary conditions for battery discharge process are summarized in Table 1 42. The governing equations, Eqs (1), (7) and (10), describe the macroscopic transport in the cell; Eq. (2) describes the microscopic diffusion of lithium in solid active material particles, as illustrated in Fig. 1. The combined micro/macroscopic model is essential to model the transport processes during lithium ion battery charge/discharge operations. Table 2 presents the values of major parameters used in the present study.

### Numerical strategy

**Figure 2:**
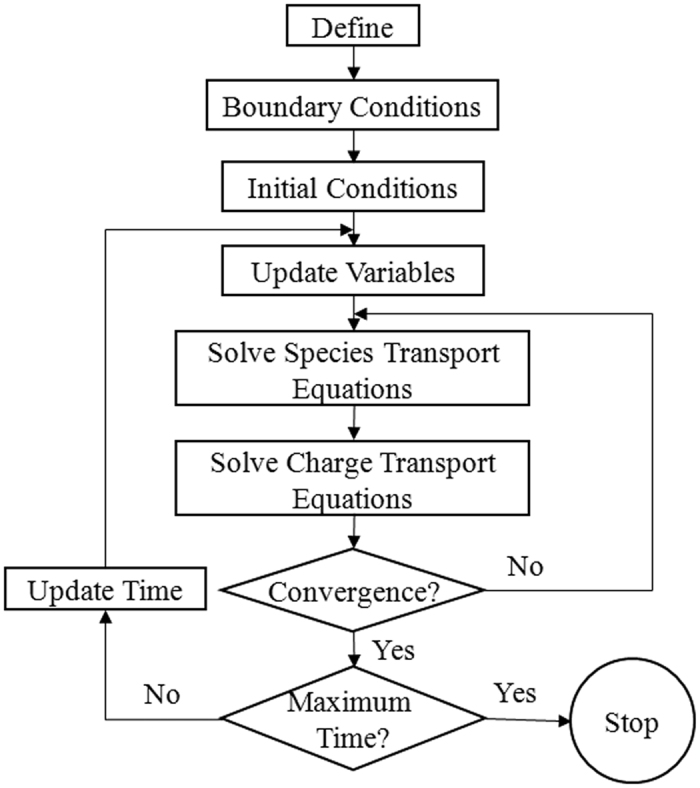
Flow chart of the computational program.

Once appropriate boundary conditions are defined, as shown in Table 1, and initial conditions are established, the governing equation group consisting of Eqs (1), (2), (7) and (10) is solved for the four unknowns: *φ*_*s*_, *φ*_*e*_, *c*_*e*_, and *c*_*s,e*_(*c*_*s,e*_ = *c*_*s*_|_*r* = *Rs*_) in the commercial computational fluid dynamics flow solver, Fluent^®^, which is based on the finite volume approximation. By customizing its User Defined Functions, various source terms and physicochemical properties are implemented. The first order upwind differencing scheme is generally used to discretize the spatial-derivative terms and a fully implicit scheme is used to discretize the transient terms. To accelerate convergence, the algebraic multi-grid iterative method is applied to solve the linearized algebraic equations. A flow chart is presented in Fig. 2 to illustrate the overall procedure of the Fluent^®^ computational program.

To examine the error of numerical discretization, the Richardson extrapolation technique^51^ was adopted. The obtained result indicates all the calculated values of the major variables deviate from the corresponding extrapolated accurate values by about 5% at most for the numerical mesh system used in the present work, approving the accuracy and fidelity of the simulation results.

The above-described electrochemical model is actually an adaption of a previous model^42^, which modeled LiFePO_4_/graphite batteries and was validated by comparing the simulated cell voltage curves with and the experimentally measured ones during battery discharge processes. Therefore, an experimental validation of the present model was omitted.

### Cases considered

We consider six cases to study the mechanisms for species and charge transport limit the performance of Li-ion batteries. The base case (i.e. case 1) has realistic battery design and is operated at normal discharge rate, 1C; the electric conductivities (*κ* and *σ* both) are specified with real values. Cases 2–4 differs from the base case due to thicker electrode design, larger size of active material particles, or being operated at a higher discharge rate. Cases 5 and 6 have an artificially-reduced electric conductivity by a reduction factor of 10 and 100, respectively. In reality, a reduced electric conductivity may be found in electrodes of decreased carbon contents or different doping strategies^52^,^53^,^54^,^55^. Main parameters of the six cases considered are summarized in Table 3.

In terms of the definition of the five parameters, i.e. Eqs (4), (5), (6), (11) and (12), we calculate their values and tabulate in Table 4. For the base case, *t*_*e*_, *t*_*s*_, and *t*_*c*_ are approximately on the same order of magnitude except the *t*_s_ in the anode, which is obviously larger than the *t*_*e*_ and *t*_*c*_; for cases 2–4, the value of one of the three characteristic times is much longer than the other two. In cases 5 and 6, the magnitudes of *R*_s_-s are artificially enlarged by a factor of 10 and 100, respectively, compared with case 1. It is worth pointing out that the magnitude of transfer current density (i.e. |*j*^*Li*^|) was approximated by assuming uniform EC reactions in electrodes when calculating the values of *t*_*c*_ tabulated in Table 4, namely,


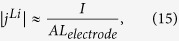


where *I* denotes the imposed current load and *A* the side surface area of the current collector plate.


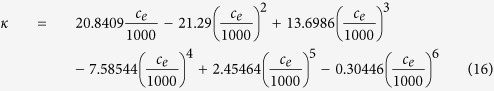



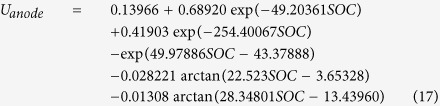



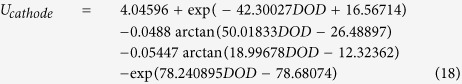


## Simulated Results and Discussion

### Base case (Case 1)

**Figure 3:**
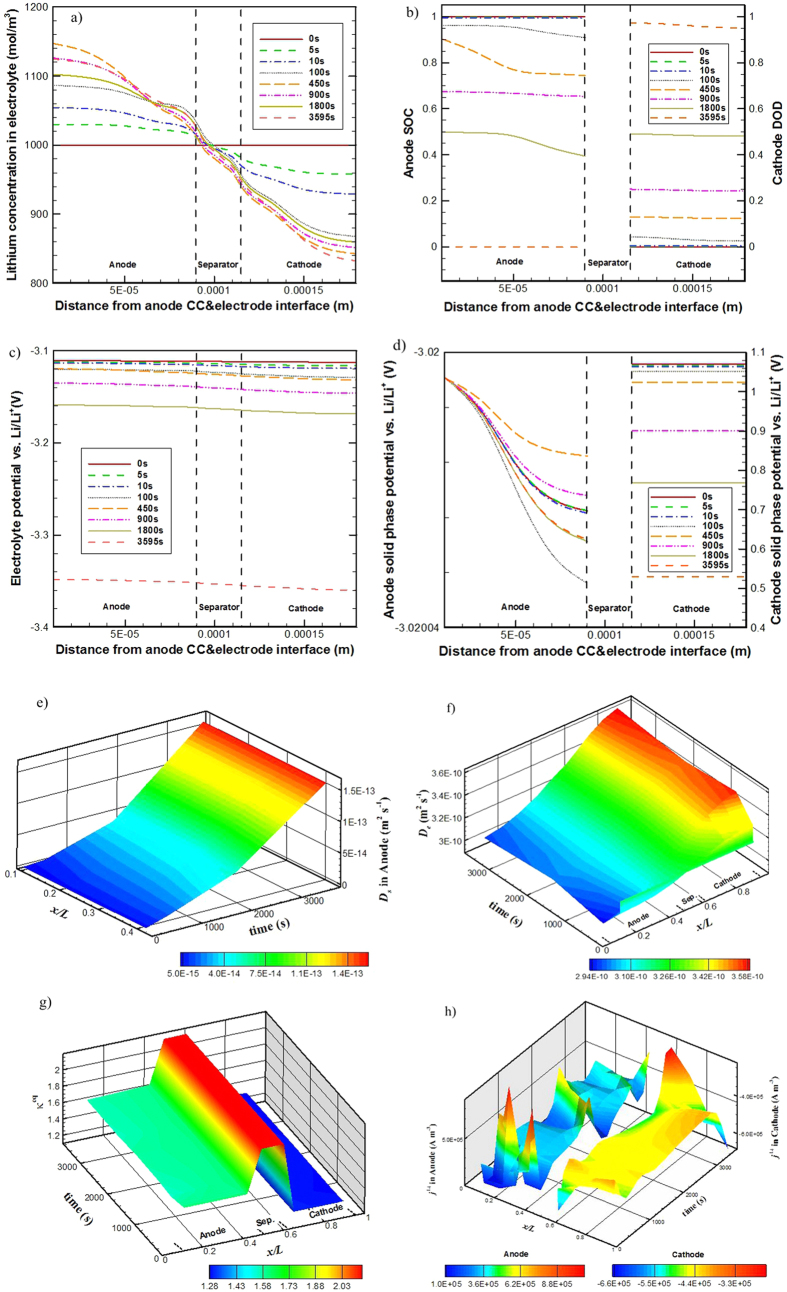
Detailed simulation results from the base case: time-variation of Li^+^ concentration distribution in electrolyte (**a**), of SOC in anode and DOD in cathode (**b**), of electrolyte phase potential distribution (**c**), and of solid phase potential distribution (**d**); spatial and temporal evolution of *D*_s_ at the surface of anode active material particles (**e**), of *D*_e_ in the electrolyte (**f**), of *κ*^eq^ (**g**), and of *j*^Li^ (**h**).

The simulated spatial distribution of Li-ion concentration (*c*_*e*_) in the electrolyte and its temporal change for the base case (i.e. case 1) are depicted in Fig. 3(a). A distribution of Li-ion concentration quickly builds up inside the cell upon discharge, forming a gradient which drives Li-ion species transport along the cell thickness direction from the anode to cathode. A maximum of 1150 mol·m^−3^ Li-ion concentration is calculated in the anode at 450 s and a minimum of 830 mol·m^−3^ Li-ion concentration is calculated in the cathode at 3595 s. Except for the very early period of the discharge process, during which the *c*_*e*_ evidently increases in the anode and decreases in the cathode with time, the distribution of *c*_*e*_ shows small variations with time. For the base case, *t*_*c*_ is larger than *t*_*e*_ as shown in Table 4, which means the consumed Li-ion in the cathode or the generated Li-ion in the anode due to local EC reactions can be supplemented or removed by the Li-ion transport process in the electrolyte. That is to say, the Li-ion transport in electrolyte does not impose any limitation to the cell discharge performance.

The time-variation of the spatial distribution of state of discharge (SOC) in the anode and depth of discharge (DOD) in the cathode for the base case are shown in Fig. 3(b). The SOC in anode and DOD in cathode are defined as


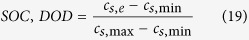


where *c*_*s,e*_ is the lithium concentration at the surface of solid active material particles; *c*_*s,max*_ and *c*_*s,min*_ are the allowed maximum and minimum concentration of lithium in the solid active material, respectively.

Seen from Fig. 3(b), the SOC decreases and the DOD increases with time as expected. Along the cell thickness direction the DOD in the cathode shows relatively more uniform distribution than the SOC in the anode; the minimum SOC appears in the near-region of the electrode/separator interface in the anode. The discharge process lasts for about 1 hour, the SOC in anode decreases from 1 to 0 and the DOD in cathode increases from 0 to 1, indicating no detectable performance limitation for the cell discharge process.

Figure 3(c) gives the spatial distribution and temporal change of the electrolyte phase potential (*φ*_*e*_) in the base case. It decreases overall with time throughout the discharge process and specifically during the late period of the process, the decrease is at a much faster rate. This is due to the diminishing open circuit potentials. Across the direction of the cell thickness, the magnitude of *φ*_*e*_ value decreases with an increase in *x* value, forming a *φ*_*e*_ gradient to drive Li-ion charge transport from the anode to cathode.

Figure 3(d) shows the spatial distribution and temporal change of the solid phase potential (*φ*_*s*_) of the base case. In the cathode, the *φ*_*s*_ value decreases overall with time throughout the discharge process; during the late period of the process, the *φ*_*s*_ is seen to decrease a lot. The value of *φ*_*s*_ in the anode overall changes little with time as a *φ*_*s*_ reference point (−3.02 V) is set in the anode current collector. In the anode across the cell thickness direction, the *φ*_*s*_ value slightly decreases with the increase of *x* and in the cathode it also slightly decreases with the increase of *x*, forming *φ*_*s*_ gradients to drive electronic charge transport from the separator side to the current collector side in the anode and from the current collector side to the separator side in the cathode. The overall decrease of *φ*_*s*_ value in the cathode is due to the decreasing open circuit potential.

*D*_*s*_is a constant value for cathode whereas in anode it is time variant; the *D*_*e*_ is a function of the *c*_*e*_. (See in Table 2) Fig. 3(e,f) give the time-variation history of *D*_*s*_ in the anode and *D*_*e*_ in the electrolyte, respectively. The *D*_*s*_ in anode increases with time, from 5.0 × 10^−15^ to 1.5 × 10^−13^ m^2^ s^−1^, substantially improving the lithium diffusion in solid active materials; the *D*_*e*_ shows relatively smaller spatial and temporal changes, within 2.9–3.6 × 10^−10^ m^2^ s^−1^. Along the cell thickness direction *D*_*s*_ in the anode shows relatively uniform distribution. Except for the very early period of the discharge process, during which the *D*_*e*_ evidently decreases in the anode and increases in the cathode with time, the distribution of *D*_*e*_ changes little with time. The value of *D*_*e*_ in the cathode is larger than that in the anode.

Figure 3(g) gives the time-variation history of *κ*^eq^. The *κ*^eq^ shows little temporal dependence, but relatively larger spatial dependence, and in the separator it takes the maximum value. The ionic conductivity *κ*, which is a function of *c*_*e*_ (refer to Eq. (16)), takes a maximum value when *c*_*e*_ = 1.0 mol∙L^−1^. From Fig. 3(a), though there is a *c*_*e*_ distribution quickly forming in the cell upon discharge, the *c*_*e*_ value in the separator region is around 1.0 mol∙L^−1^ all the time through the discharge process. This adequately explains the spatial dependence of *κ*^eq^ and why the *κ*^eq^ is seen to take the maximum value in the separator region.

Figure 3(h) gives the spatial and temporal evolution of *j*^Li^, the magnitude of which reflects the electro-chemical reaction intensity. Upon discharge, the EC reactions occur and a transfer current density distribution quickly builds up in both the electrodes. In the anode, the transfer current density is positive while in the cathode is negative. Overall, the EC reaction in the cathode is more uniform than that in the anode. In the anode, the transfer current density fluctuates with time and the location with stronger EC reaction shifts frequently. In the cathode, the near-region of separator is seen to have slightly stronger EC reaction for most time of the discharge process, though some small fluctuations at the location of stronger EC reaction are also seen; in the late period of the discharge process, after ~3500 s, the near-region of cathode current collector has distinctly stronger EC reaction.

Table 5 summarizes the time-variation of the five characteristic parameters. During the discharge process, the variation of *t*_*e*_ is small due to the small spatial and temporal changes of the *D*_*e*_. (Refer to Fig. 3(f)). The *t*_*s*_ in anode remarkably decreases with time due to the substantial increase of *D*_*s*_ during the discharge process. (See Fig. 3(e)) The spatial and temporal changes of *j*^Li^ and *c*_*e*_ (See Fig. 3(a,h)) leads to the time-variation of *t*_*c*_, which are not large for this particular case. In the early period of the discharge process the *t*_s_ in the anode is obviously larger than the *t*_*e*_ and *t*_*c*_. However, the diffusion of lithium in the anode active material particles should occur only in the particle’s near-surface region during the early period of discharge; calculating the *t*_s_ in terms of the characteristic size parameter *r*_0_ overestimates the real characteristic time for the lithium diffusion in anode active material particles. The *t*_s_ in the anode is largely reduced with the progress of discharge; after about 1000 s discharge, the *t*_s_ in the anode turns to be on the same order of magnitude of the *t*_*e*_ and *t*_*c*_. Therefore, the cell is fully discharged without any capacity loss.

Seen from Table 5 also, the *R*_*s*_is constant and the *R*_*e*_ shows small changes with time due to the spatial and temporal changes of *κ*^eq^ (see Fig. 3(g)). Detailed discussion about the charge transport effects on the cell performance will be presented in Section 4.5.

### Thick electrode case (Case 2)

**Figure 4:**
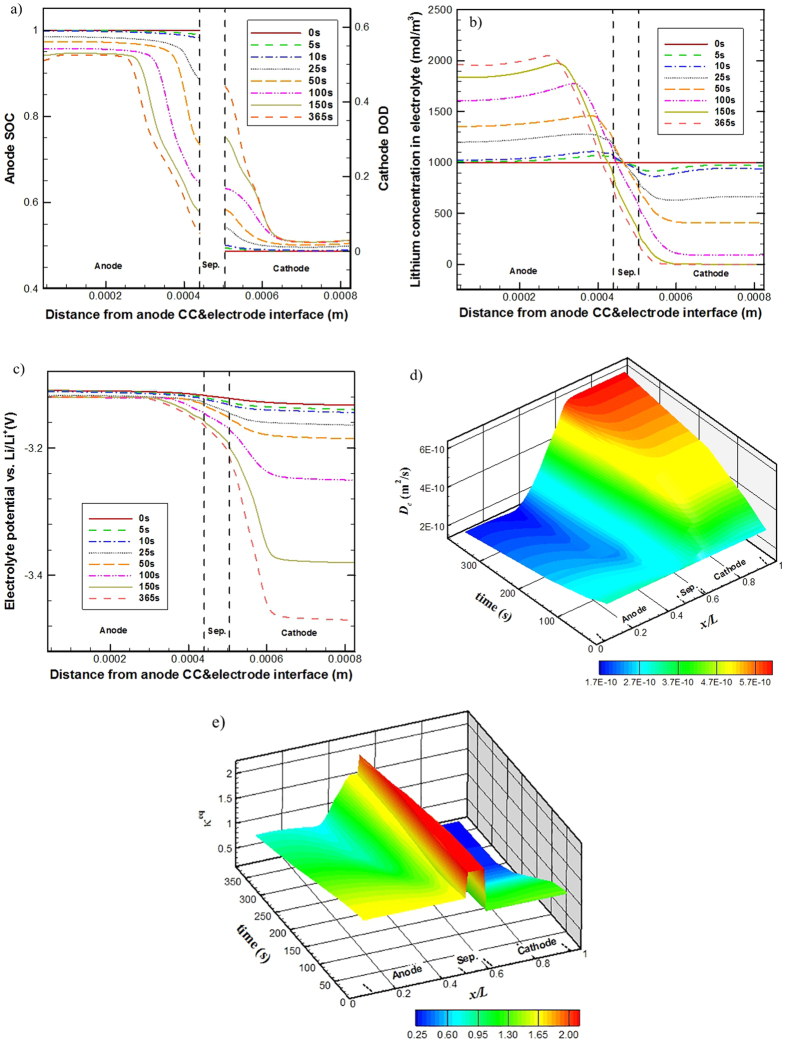
Part of simulation results from case 2: time-variation of SOC in anode and DOD in cathode (**a**), of Li^+^ concentration distribution in electrolyte (**b**), and of electrolyte phase potential distribution (**c**); spatial and temporal evolution of *D*_e_ in the electrolyte (**d**), and of *κ*^eq^ (**e**).

Increasing the thickness of electrodes might be an effective way to simultaneously enhance the energy density and power density both of Li-ion batteries^40^. Case 2 considers a cell, in which the electrode thickness is five times of the base case for both anode and cathode side. The simulated spatial distribution and temporal change of SOC in the anode and DOD in the cathode for case 2 are shown in Fig. 4(a). The SOC decreases and the DOD increases with time during the discharge process. The SOC in anode and the DOD in cathode both show very large non-uniform profiles. The discharge process terminates at about 365 s, which is far below the 1C discharge time, 3600 s. The *t*_*e*_ is approximately proportional to the square of electrode thickness. (See Eq. (4)) The 4-times increased electrode thickness of case 2 largely increases the value of *t*_*e*_ (see in Table 4), which causes severe capacity loss, only about 10% of the stored electricity can be discharged; a large portion of the electrodes is actually not in use.

Figure 4(b) shows the spatial distribution and temporal change of Li-ion concentration (*c*_*e*_) in electrolyte for case 2. The *c*_*e*_ value increases in the anode and decreases in the cathode throughout the discharge process. A maximum of 2100 mol·m^−3^ Li-ion concentration is calculated in the anode and a minimum of 0 mol·m^−3^ Li-ion concentration is calculated in the cathode at the end of discharge process. The zero *c*_*e*_ in the near-region of the cathode current collector indicates that the lithium ion is depleted in the electrolyte, which is due mainly to the too slow Li-ion transport speed (i.e. too large *t*_*e*_) in the electrolyte.

From Table 2, for case 2 it is clearly shown that the *t*_*e*_ is much larger than the *t*_*c*_-s, which means in the electrolyte the Li-ion consumption/generation speed due to local EC reactions in the cathode/anode cannot be supplemented/removed in time by the Li-ion transport in the electrolyte phase. Therefore, we see in Fig. 4(b) very large value of *c*_*e*_ and locally zero *c*_*e*_ value present in the anode and cathode, respectively.

Figure 4(c) gives the spatial distribution and temporal change of the electrolyte phase potential (*φ*_*e*_) for case 2. The *φ*_*e*_ value overall decreases with time throughout the discharge process. A zero *φ*_*e*_ gradient appears in the near-regions of the two current collectors, indicating actually no ionic charge transport presents in these regions.

Figure 4(d) presents the time-variation history of *D*_*e*_ distribution in the electrolyte for case 2. *D*_*e*_ value decreases in anode and increases in cathode with time. For case 2, the distribution of *D*_*e*_ shows larger spatial and temporal dependence than the base case as the *c*_*e*_ distribution for case 2 tends to establish larger gradients across the separator along the electrode through-plane direction (refer to Figs 3(a) and 4(b)).

Figure 4(e) gives the time-variation history of the *κ*^eq^ distribution in the electrolyte phase for case 2. The *κ*^eq^ result for case 2 shows larger spatial and temporal changes compared with the result of the base case given in Fig. 3(g). Due to the resultant zero *c*_*e*_ in cathode, the *κ*^eq^ for case 2 even shows very low (close to zero) *κ*^eq^ in the near-region of the cathode current collector during the late period of the discharge process. This leads to a very large ionic charge transport resistance, making the discharge operation actually not viable in this region.

Table 6 summarizes the time-variation of the *t*_*e*_, *t*_*s*_, and *t*_*c*_ for case 2. The *t*_*e*_ and *t*_*c*_-s all show larger amounts of changes than those for the base case (see Table 5), whereas the *t*_*s*_ in anode shows smaller amounts of increase due to the incomplete Li-insertion process. For case 2 itself, the time-variation of *t*_*e*_ is not that significant as that of *t*_*c*_ in cathode, and the *t*_*e*_ remains always much larger than the *t*_*s*_ in cathode and the *t*_*c*_-s. The largely reduced *c*_*e*_ and the locally increased transfer current density in the cathode owing to partially cease-operation of the cell dramatically shorten the value of the *t*_*c*_ in cathode, which combines the too slow Li-ion transport speed to lead to a quick termination of the discharge process.

### High rate discharge case (Case 3)

**Figure 5:**
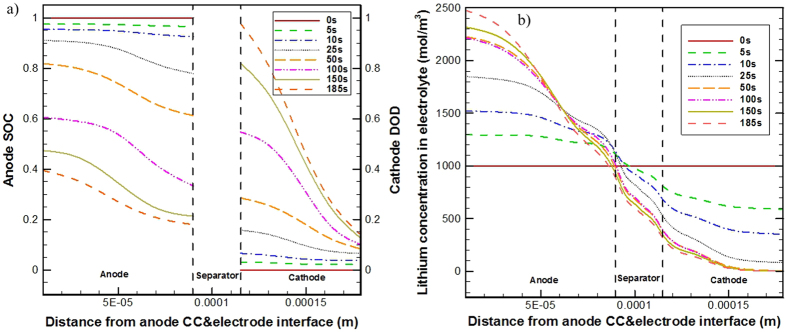
Time-variation of SOC in anode and DOD in cathode (**a**), and of Li^+^ concentration distribution in electrolyte (**b**) for case 3.

The practical uses of various lithium-ion batteries of different capacities often require the batteries being of adequate high-rate charge/discharge capability^24^. Case 3 considers a 10 C discharge process. The simulated spatial distribution and temporal change of SOC in the anode and DOD in the cathode are depicted in Fig. 5(a). The SOC decreases and the DOD increases with time during the discharge process. The SOC in anode and the DOD in cathode both show very large non-uniform profiles. The discharge process terminates at about 185 s, which is about half of the 10 C discharge time, meaning only about 50% of the stored electricity is discharged. The *t*_*c*_ is inversely proportional to the discharge current. The 10-times increased current load greatly shortens the value of *t*_*c*_ (see in Table 4), leading to too fast speed of Li-ion consumption/generation owing to the EC reactions in the cathode and anode, respectively.

Figure 5(b) gives the spatial distribution and temporal change of Li-ion concentration (*c*_*e*_) in the electrolyte for case 3. The *c*_*e*_ increases overall in the anode and decreases in the cathode with time. The large current density renders a *c*_*e*_ profile of large difference along the electrode through-plane direction. A maximum of 2500 mol·m^−3^ Li-ion concentration is calculated in the anode and a minimum of 0 mol·m^−3^ Li-ion concentration is calculated in the cathode at the end of discharge process. The zero value of *c*_*e*_ in the near-region of the cathode current collector indicates that the Li-ion in the electrolyte is depleted. The discharge process terminates at 185 s, much less than the 10 C discharge time, 360 s, signifying again severe capacity loss.

Table 7 summarizes the time-variation of the *t*_*e*_, *t*_*s*_, and *t*_*c*_. Compared with the base case (see Table 5), the *t*_*e*_ and *t*_*c*_ for case 3 both show larger amount of changes, whereas the *t*_*s*_ in anode shows smaller amount of changes due to the incomplete Li-insertion process. For case 3 itself, the *t*_*c*_-s remain always much smaller than the *t*_*s*_ in anode and the *t*_*e*_; the time-variation of *t*_*c*_ in the cathode is very significant, it is shortened dramatically due to the largely reduced *c*_*e*_, exacerbating the performance-limitation condition and finally leading to a quick termination of the discharge process.

It has been shown previously^37^ that high-rate discharges of Li-ion batteries are limited by species transport processes, which can be the Li-ion species transport in the electrolyte phase or the lithium transport in the solid active material phase or the both. However, it is still not clear which factor is the main limitation mechanism to the high-rate discharges of Li-ion batteries. From the above-detailed analysis of case 3 in the present work, the performance limitations of high-rate charge/discharge processes are essentially due to the very short *t*_*c*_, i.e. the too fast depletion of Li-ions in electrolyte phase due to the EC reaction.

### Case with active materials of larger particle size (Case 4)

**Figure 6:**
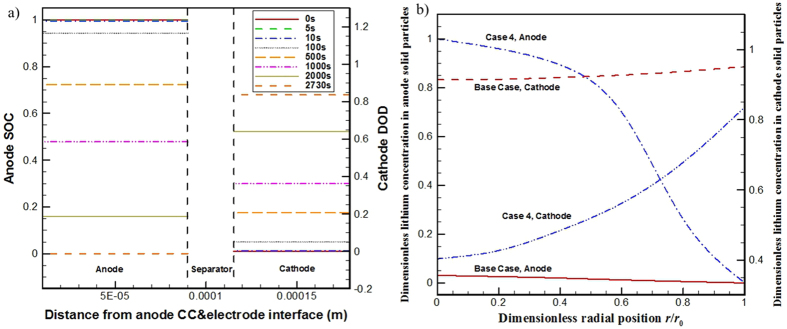
(**a**) Time-variation of the SOC in anode and the DOD in cathode for case 4, and (**b**) the lithium concentration in solid active particles at the end of discharge process for the base case and case 4.

Case 4 considers active material particles of larger sizes, in anode and cathode both the active material particle size is increased by a factor of 4. As the *t*_*s*_ is directly proportional to the squared particle radius, it is 25-times enlarged. The simulated spatial distribution and temporal change of SOC in the anode and DOD in the cathode for case 4 are presented in Fig. 6(a). The SOC in anode is reduced down to zero whereas the DOD in cathode is raised up to about 0.82 at 2730 s. The zero SOC in anode indicates the end of the discharge process and the operation duration (i.e. 2730 s) indicates only about 75.8% of the stored electricity is discharged, meaning marginal loss at the cell capacity. The *t*_*s*_-s are far more than the *t*_*c*_-s and *t*_*e*_ as seen in Table 4, which means the lithium diffusion in the active materials cannot transport the lithium into the particle interior or out from the particle interior to the particle surface in time. It is the slow lithium diffusion speed in the solid active material particles that limits the cell discharge performance.

For case 4, the SOC in anode can be reduced down to zero, indicating seemingly that the cell can be fully discharged. We examined the lithium concentration in the solid active particles at the end of the discharge process, 2730 s. The simulated dimensionless lithium concentration distributions in both electrodes are displayed in Fig. 6(b). The displayed lithium concentration values are non-dimensionalized in terms of Eq. (19). Due to the too slow lithium diffusion speed in solid active materials, there exists large lithium concentration difference in the solid active material particles for both electrodes. At the end of discharge operation the dimensionless lithium concentration at the anode solid active particle surface is about 0, which is the minimum value it can reach, whereas it remains about 1 (i.e. unchanged from the beginning of the discharge process) at the center of the particle; in the cathode, the situation looks better, the dimensionless lithium concentration at the solid active material particle surface rises to about 0.8 while it is only about 0.4 at the center of the particle. It is the zero dimensionless lithium concentration at the solid anode active material particle surface that leads to the termination of the discharge process.

For comparison, the dimensionless lithium concentration distributions in solid active material particles at the end of the discharge process for the base case are also plotted in Fig. 6(b). Contrast to case 4, for the base case, very small differences of dimensionless lithium concentration are seen in the solid active material particle for both electrodes, indicating the lithium diffusion processes in solid active materials are fast enough and will not impose any limitations to the cell discharge performance.

Table 8 summarizes the time-variation of the *t*_*e*_, *t*_*s*_, and *t*_*c*_ for case 4. The *t*_*s*_ in anode decreases with time but is always larger the *t*_*s*_ in cathode; the *t*_*e*_ and *t*_*c*_-s all slightly change with time due to the spatial and temporal changes of *D*_e_, *c*_e_ and *j*^Li^. However, the *t*_*s*_-s in anode and cathode both are always at least one order of magnitude larger than the *t*_*e*_ and the corresponding *t*_*c*_, meaning the cell performance is limited by the lithium diffusion processes in solid active materials. Moreover, as the *t*_*s*_ in cathode is relatively shorter than that in the anode, the termination of the discharge process is mainly due to the depletion of lithium in the near-surface region of anode solid active material particles. (Refer to Fig. 6(b))

### Effects of charge transport

During charge/discharge of Li-ion batteries, the ionic charge is transporting in the electrolyte phase between the two electrodes across the separator and the electronic charge is transporting in the solid phase of electrodes. Theoretically, the ionic and electronic charge transports will always seek pathways of the least total resistance, which affects local EC reaction intensity (i.e. the magnitude of *j*^Li^) in the anode and cathode electrodes. Qualitatively, if the *R*_*e*_, defined by Eq. (11), is greater than the *R*_*s*_, defined by Eq. (12), the EC reaction will tend to occur in the near-region of the separator for anode and cathode both to reduce the ionic charge transport resistance in the electrolyte, otherwise, it will tend to occur in the near-region of the current collector for anode and cathode both to reduce the electronic charge transport resistance in the electrodes. As the overpotential (*η*), which is the driving force of EC reactions, is linearly dependent on the open-circuit potential (*U*_a_ in anode or *U*_c_ in cathode), one other factor that affects or even dominates the EC reaction distribution in electrodes is the open-circuit potentials. The *U*_a_ is a function of the SOC in anode and the *U*_c_ is a function of the DOD in cathode, as defined by Eqs (17) and (18), respectively.

**Figure 7:**
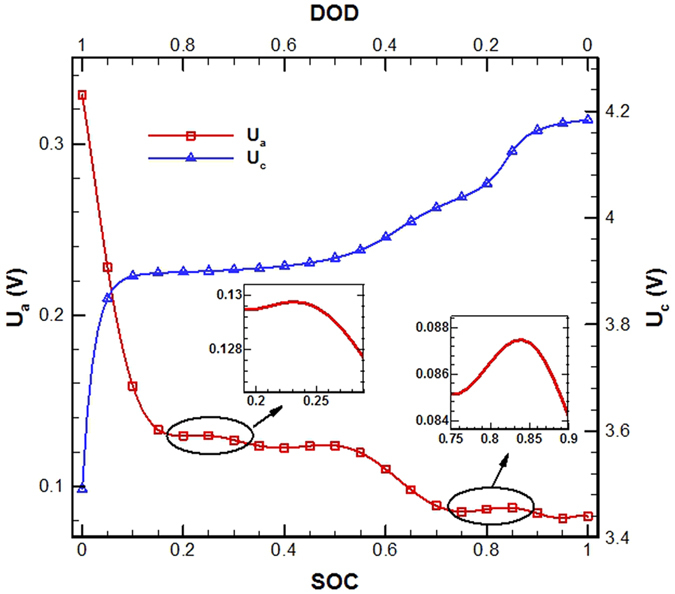
Open circuit potential of anode (Li*x*C_6_), U_a_, and cathode (Li*y-x*CoO_2_), U_c_.

Seen from Tables 4 and 5, for the base case, the *R*_*e*_ in anode is about 4.9 × 10^−5^ Ω, much larger than the *R*_*s*_, 1.7 × 10^−6^ Ω; the *R*_*e*_ in cathode is about 4.9 × 10^−5^ Ω, larger than but still on the same order of magnitude of the *R*_*s*_, 1.0 × 10^−5^ Ω. The *R*_*e*_ and *R*_*s*_ are both very small and only lead to very small *φ*_*s*_ and *φ*_*e*_ differences, about a few millivolt *φ*_*e*_ difference through the cell, about 0.03 millivolt *φ*_*s*_ difference in the anode, and about 0.1 millivolt *φ*_*s*_ difference in the cathode (Refer to Fig. 3(c,d)). Figure 7 depicts the curves of open-circuit potential versus DOD in the cathode or SOC in the anode. When discharging, the *U*_c_ decreases with the increasing DOD and the *U*_a_ increases overall with the decreasing SOC though some fluctuations are seen. However, the locally magnified images in Fig. 7 clearly indicate that even the amplitudes of fluctuations at *U*_a_ are at least a few millivolts larger than the resultant *φ*_*s*_ and *φ*_*e*_ differences in the cell. Therefore, for the base case, the EC reactions should be dominated more by the spatial and temporal changes of *U*_a_ and *U*_c_. This well explains the observations from Fig. 3(h), which presents the spatial and temporal evolution of *j*^Li^ in the two electrodes for the base case.

**Figure 8:**
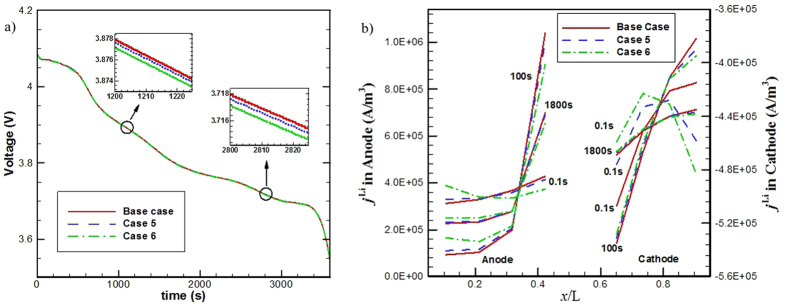
(**a**) Voltage curves of the base case, case 5 and case 6, and (**b**) comparison of *j*^Li^ profiles of the base case, case 5 and case 6 at three typical time instants.

To further clarify the effects of charge transport on the cell charge/discharge performance, two more cases (cases 5 and 6) were simulated. Case 5 considers a 10-times reduced electric conductivity and case 6 a 100-times reduced electric conductivity compared with the base case. Figure 8(a) gives the obtained output cell voltage curves for the base case, case 5, and case 6, respectively. The three curves vary almost in the same trace; the locally magnified insets show that there exist small differences among them. The base case outputs the highest cell voltage, and case 6 the lowest, but the difference between them is very small, about 1 mV.

Figure 8(b) compares the *j*^Li^ profiles of the base case, case 5 and case 6 at three typical time instants: 0.1 s, 100 s and 1800 s. Increasing the *R*_*s*_ effectively adjusts the *j*^Li^ distribution in both electrodes; the EC reaction tends to more preferably occur in the near-region of the current collector for anode and cathode both.

**Figure 9:**
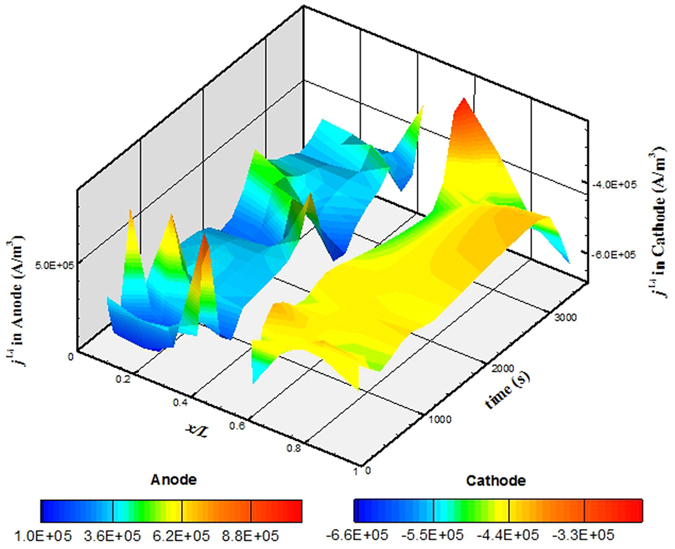
Spatial and temporal evolution of *j*^Li^ in the cell when discharging for case 6.

For case 6, the *R*_*s*_ is 1.7 × 10^−4^ Ω in anode and 1.0 × 10^−3^ Ω in cathode, both 100 times larger than that for the base case. Figure 9 presents the simulated spatial and temporal evolution of *j*^Li^ in the two electrodes. Compared with Fig. 3(h), which displays the results from the base case, the *j*^Li^ in Fig. 9 shows qualitatively similar variation trends, but the variation range is about 15% narrower for both electrodes. The increased *R*_*s*_-s make the EC reactions more uniformly occur in the electrodes.

## Conclusions

To understand the mechanisms of Li-ion battery performance being limited by species and charge transport, the present work established a general characteristic parameter analysis method. Five parameters were proposed to characterize the involved species and charge transport during battery charge/discharge processes. The first three are characteristic time parameters, namely: (1) *t*_*e*_, which characterizes the Li-ion transport rate in the electrolyte phase, (2) *t*_*s*_, characterizing the lithium diffusion rate in the solid active materials, and (3) *t*_*c*_, describing the local Li-ion depletion rate in electrolyte phase at the electrolyte/electrode interface due to EC reactions. The other two are electric resistances: *R*_e_ and *R*_s_, which represent the equivalent ionic transport resistance and the effective electronic transport resistance in the electrode, respectively.

We then established a three-dimensional electrochemical model for the graphite/LiPF_6_/LiCoO_2_ battery and simulated its discharge processes with various battery designs or operational parameters, including thick and thin electrode design, high and low discharge rate, large and small active material particles in electrodes, and high and low electric conductivity in the solid phase. Carefully analyzing the simulation results corroborates that so long as the three characteristic time parameters are on the same order of magnitude, the battery may not get into any performance-limitations caused by the species transport processes. The simulation results unravel that the performance-limitation of thick electrode battery is essentially due to the too slow Li-ion species transport in-between the two electrodes across the separator and the performance-limitation of batteries at high-rate operations is essentially due to the very fast local depletion of Li-ions in the electrolyte at the interface of solid active materials and electrolyte by the EC reaction. The simulation results indicate as well that the battery with very large active material particles in electrodes may have too large *t*_*s*_, making the battery performance limited by the too slow lithium diffusion from solid active material particle interior to its surface or the inverse.

The *R*_e_ and *R*_s_ are defined for both anode and cathode. Theoretically, the charge transports including the ionic and electronic charge transport will always seek transport paths of the least total transport resistance. That is to say, the relative magnitude of *R*_e_ and *R*_s_ will affect the *φ*_s_ and *φ*_e_ distributions in the corresponding electrode. Numerical simulations reveal that increasing the relative magnitude of *R*_s_ to *R*_e_ makes the EC reaction tend to occur in the near-region of current collector for anode and cathode both, leading to more uniform EC reactions in the electrodes.

The characteristic parameter analysis method established in the present work may be applicable to or adaptable for use in other similar electrochemical systems that involve species and charge transports, such as the electrolyser, photoelectrochemical system, solar cell and various fuel cells. Caution must be paid to the different species transport mechanisms involved in different electrochemical systems. We take the H_2_/O_2_ proton exchange membrane fuel cell (PEMFC) as an example. The transport of reactants (i.e. H_2_ and O_2_) in PEMFC relies not only on molecular diffusion like the lithium species transport in solid active particles considered in the present work, but also on fluid convection, the characteristic time parameters that describe H_2_ and O_2_ transports must have different definitions to that for the lithium species transport in solid active particles in lithium-ion batteries. Future work may expend efforts on studying the transferability of the present method to other similar electrochemical systems.

## Additional Information

**How to cite this article**: Jiang, F. and Peng, P. Elucidating the Performance Limitations of Lithium-ion Batteries due to Species and Charge Transport through Five Characteristic Parameters. *Sci. Rep*. **6**, 32639; doi: 10.1038/srep32639 (2016).

## Figures and Tables

**Table 1:** Governing equations and boundary conditions (for discharge) for the cases considered.

	Conservation equations	Boundary conditions
Species, electrolyte phase	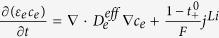 (1)	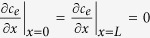 ; Zero flux at other boundaries
Species, solid phase	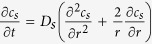 (2)	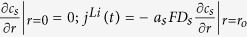
Charge, electrolyte phase	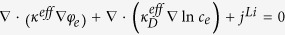 (7)	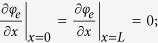 Zero flux at other boundaries
Charge, solid phase	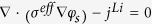 (10)	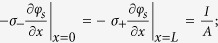 Zero flux at other boundaries

**Table 2:** Model parameters.

Parameters	Cu current collector	Anode	Separator	Cathode	Al current collector
Thickness^a^ (Base case), L (μm)	10	80	25	64	10
Particle radius^a^ (Base case), r_0_ (μm)		10	N/A	8	
Porosity^a^, ε		0.35	0.42	0.3	
Specific area in electrode^a^ (Base case), a_s_ (m^2^ m^−3^)		1.79E5		2.35E6	
Maximum Li^+^ concentration in solid^a^ (mol m^−3^)		28555	N/A	51555	
Initial electrolyte concentration^a^, c_e_ (mol m^−3^)		1000			
Reference exchange current density^56^, *i*_0_ (A m^−2^)		36		26	
Solid phase electronic conductivity, σ (S m^−1^)	6.0E7^57^	100.0^56^	0	10^56^	3.8E7^56^
Ionic conductivity in electrolyte^a^, κ (S m^−1^)	0	Eq. (16)			0
Li^+^ diffusion coefficient in electrolyte^58^, D_e_ (m^2^ s^−1^)	0	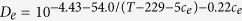			0
Li diffusion coefficient in solid, D_s_ (m^2^ s^−1^)		*D*_s_ = 3.9 × 10^−14^(1.5-SOC)^3.5^ 59	N/A	1.0E-13^56^	
Surface overpotential, *η* (V)		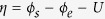			
Open circuit voltage, *U* (V)^56^		Eq. (17)		Eq. (18)	
mean molar activity coefficient of the electrolyte, *f*_±_^60^		1			
Anodic/Cathodic transfer coefficient^56^, α_a,_ α_c_		0.5		0.5	
Transference number^56^, 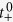	0.363				
Faraday’s constant, F (C mol^−1^)	96487.0				

^a^Data from our industrial partner: Amperex Technology Limited.

**Table 3:** Simulated cases.

Case #	Thickness (μm)		Particle size (μm)		Electric conductivity in electrodes	Discharge rate
	Anode	Cathode	Anode	Cathode		
Case 1 (Base case)	80	64	10	8	*κ*, *σ*	1C
Case 2	400	320	10	8	*κ*, *σ*	1C
Case 3	80	64	10	8	*κ*, *σ*	10C
Case 4	80	64	40	32	*κ*, *σ*	1C
Case 5	80	64	10	8	*κ*, *σ*/10	1C
Case 6	80	64	10	8	*κ*, *σ*/100	1C

**Table 4:** Values of the five characteristic parameters when the battery is in the idle state prior to discharge.

Case #	*t*_e_ (s)	*t*_s_ (s)		*t*_c_ (s)c1		*R*_e_ (Ω)c2		*R*_s_ (Ω)c2	
		Anode	Cathode	Anode	Cathode	Anode	Cathode	Anode	Cathode
Case 1	180.4	3.2E3	71.1	147.7	101.3	4.9E-5	4.9E-5	1.7E-6	1.0E-5
Case 2	4.4E3	3.2E3	71.1	147.7	101.3	2.5E-4	2.5E-4	8.2E-6	5.0E-5
Case 3	180.4	3.2E3	71.1	14.8	10.1	4.9E-5	4.9E-5	1.7E-6	1.0E-5
Case 4	180.4	5.2E4	1.1E3	147.7	101.3	4.9E-5	4.9E-5	1.7E-6	1.0E-5
Case 5	180.4	3.2E3	71.1	147.7	101.3	4.9E-5	4.9E-5	1.7E-5	1.0E-4
Case 6	180.4	3.2E3	71.1	147.7	101.3	4.9E-5	4.9E-5	1.7E-4	1.0E-3

^c1^|*j*^*Li*^| is calculated by Eq. (15).

^c2^The side surface area of electrodes takes the value of 1 m^2^.

**Table 5:** Time-variation of the five characteristic parameters during discharge for the base case.

Time instant (s)	*t*_e_ (s)	*t*_s_ (s)		*t*_c_ (s)		*R*_e_ (Ω)c2		*R*_s_ (Ω)c2	
		Anode	Cathode	Anode	Cathode	Anode	Cathode	Anode	Cathode
10	178.3–182.0	3.1E3–3.1E3	71.1	122.0–184.0	69.6–124.3	4.9E-5	4.9E-5–5.0E-5	1.7E-6	1.0E-5
100	177.0 –183.2	1.8E3–2.5E3		53.6–618.8	80.0–103.2	4.9E-5–5.0E-5	5.0E-5		
500	174.0–187.5	7.5E2–1.2E3		66.7–351.4	80.4–101.0	4.9E-5–5.0E-5	5.0E-5		
1000	175.2–185.2	4.7E2–5.0E2		158.4–163.1	87.8–91.9	4.9E-5–5.0E-5	5.0E-5		
1800	176.2–184.1	2.0E2–2.8E2		79.2–257.4	89.7–91.0	4.9E-5–5.0E-5	5.0E-5		
2400	174.5–186.3	1.5E2–1.7E2		103.5–296.4	83.5–93.2	4.9E-5–5.0E-5	5.0E-5		
3595	174.9–185.4	68.9–69.0		83.1–262.1	56.9–174.3	4.9E-5–5.0E-5	5.0E-5		

^c2^The side surface area of electrode takes the value of 1 m^2^.

**Table 6:** Time-variation of the *t*_*e*_, *t*_*s*_, and *t*_*c*_ during discharge for case 2.

Time instant (s)	*t*_e_ (s)	*t*_s_ (s)		*t*_c_ (s)	
		Anode	Cathode	Anode	Cathode
5	4.3E3–4.5E3	3.0E3–3.2E3	71.1	52.3–388.9	28.9–195.5
10	4.3E3–4.5E3	2.9E3–3.2E3		57.5–283.4	27.8–163.5
50	4.3E3–4.6E3	1.6E3–2.9E3		25.3–372.3	22.0–123.1
100	4.2E3–4.9E3	724.2–2.7E3		37.5–560.2	19.4–108.5
180	3.9E3–5.4E3	522.7–2.5E3		55.5–778.8	7.9–56.1
240	3.6E3–5.7E3	445.6–2.3E3		72.1–1.0E3	1.7–21.6
365	3.2E3–6.0E3	315.3–2.2E3		39.8–3.0E3	0.4–30.7

**Table 7:** Time-variation of the *t*_*e*_, *t*_*s*_, and *t*_*c*_ during discharge for case 3.

Time instant (s)	*t*_e_ (s)	*t*_s_ (s)		*t*_c_ (s)	
		Anode	Cathode	Anode	Cathode
5	173.5–189.5	2.6E3–2.7E3	71.1	13.2–23.2	5.5–7.6
10	169.3–202.4	2.0E3–2.4E3		11.5–33.8	3.8–5.7
50	149.6–274.2	432.4–1.1E3		15.2–38.1	0.2–1.6
100	152.6–272.8	165.8–419.8		10.4–45.1	0.1–1.7
120	150.0–272.1	132.9–337.9		14.9–46.7	0.1–1.5
160	144.3–296.7	113.8–236.9		18.1–40.1	0.1–1.1
185	142.7–417.5	102.8–175.6		16.7–31.2	0.0–0.9

**Table 8:** Time-variation of the *t*_*e*_, *t*_*s*_, and *t*_*c*_ during discharge for case 4.

Time instant (s)	*t*_e_ (s)	*t*_s_ (s)		*t*_c_ (s)	
		Anode	Cathode	Anode	Cathode
10	180.1–180.2	5.0E4	1.1E3	155.2	94.3
100	180.3–180.5	3.6E4		163.3	87.1
500	180.3–180.5	1.1E4		163.3	87.1
1000	180.3–180.5	4.2E3		163.3	87.1
1800	180.3–180.5	1.9E3		163.3	87.1
2400	180.3–180.5	1.3E3		163.3	87.1
2730	180.3–180.5	1.1E3		163.3	87.1
